# The impact of mission valence on innovative work behavior among civil servants: the roles of harmonious passion, spiritual leadership, and the organization’s fault-tolerant climate

**DOI:** 10.3389/fpsyg.2025.1722923

**Published:** 2025-11-13

**Authors:** Xi Liu, Shaobo Zhang, Qian Huang

**Affiliations:** 1 College of Public Administration, Huazhong University of Science and Technology, Wuhan, China; 2 Industrial Culture Development Center, Ministry of Industry and Information Technology, Beijing, China; 3 School of Smart Sports Engineering, Wuhan Sports University, Wuhan, China

**Keywords:** mission valence, innovative work behavior, harmonious passion, organization’s fault-tolerant climate, spiritual leadership

## Abstract

**Background:**

Civil servants’ innovative work behavior is crucial for government transformation and the improvement of public service efficiency, especially in developing and transitional countries. However, the pathways to effectively motivate innovative work behavior among civil servants remain to be further explored in academic research.

**Objective:**

This study, grounded in the Conservation of Resources Theory and the Broaden-and-Build Theory of Positive Emotions, aims to explore whether and how mission valence affects the innovative work behavior of civil servants in China. Specifically, this research constructs a moderated mediation model to examine the mediating role of harmonious passion, as well as the moderating effects of spiritual leadership and the organization’s fault-tolerant climate.

**Materials and methods:**

This study collected valid matched data from 456 civil servants in China through a two-wave survey. We conducted reliability and validity analysis, correlation analysis, hierarchical regression, and Bootstrapping tests using SPSS 27.0 and AMOS 24.0 to validate the proposed theoretical model.

**Results:**

The empirical results indicate that: (1) Mission valence has a significant positive effect on civil servants’ innovative work behavior; (2) Harmonious passion partially mediates the relationship between mission valence and innovative work behavior of civil servants; (3) Spiritual leadership positively moderates the relationship between mission valence and harmonious passion of civil servants; (4) Organization’s fault-tolerant climate positively moderates the relationship between harmonious passion and innovative work behavior, and also positively moderates the mediating effect of harmonious passion.

**Conclusion:**

This study deepens the understanding of the relationship between mission valence and innovative work behavior of civil servants in the Chinese context. The findings provide practical insights for public sector managers to develop effective strategies, such as strengthening organizational mission promotion, fostering harmonious passion, advocating spiritual leadership, and cultivating a fault-tolerant climate, to enhance civil servants’ innovative work behavior.

## Introduction

In the context of the ongoing global wave of government reforms, civil servants are tasked with driving the process of government reform, improving government governance performance, and effectively responding to citizens’ needs. They play a crucial role in advancing government transformation and development (Vigoda, 2000; Graves et al., 2021; Congo and Choi, 2022). In recent years, although governments at various levels have devoted considerable efforts to enhancing civil servants’ capacity for innovation and social governance, the overall work enthusiasm within the civil service still faces serious challenges in practice. Confronted with intensified power supervision, a narrowing space for accountability, a high volume of repetitive tasks, and limited promotion opportunities, some civil servants have experienced varying degrees of job burnout (Miao et al., 2024), resulting in diminished enthusiasm and proactivity at work. This has seriously constrained the effective provision of public services and hindered the rapid advancement of social governance. This has severely restricted the efficient provision of public services and the rapid development of social governance. The innovative work behavior of civil servants is a crucial way for them to contribute to government organizational reforms. It can optimize the quality of public services, improve the government-citizen interaction, and enhance the efficiency of government operations (Mehmood et al., 2022). Therefore, how to effectively stimulate the innovation of civil servants is one of the key issues that need to be addressed in the current human resource management practices of the public sector. Existing research suggests that organizational-level factors such as institutional environment, leadership style, and organizational climate significantly affect the innovative work behavior of civil servants (Bak et al., 2022; Vassallo et al., 2023; Lee and Jung, 2025). In addition, some studies have found that individual-level factors, such as public service motivation, play an important role in promoting the innovative work behavior of civil servants (Nguyen et al., 2023). However, there is still room for further exploration in academia regarding the impact of individual cognitive factors on the innovative work behavior of civil servants.

Compared to the private sector, the public sector is relatively limited in terms of its incentive mechanisms (Shen et al., 2016). This is specifically manifested in the following: First, the extrinsic nature of innovative work behavior makes it difficult to effectively incorporate it into the performance evaluation system. Second, it is difficult to stimulate the innovative work behavior of civil servants through flexible methods such as salary adjustments and promotions. As a result, researchers have shifted their focus to the organizational mission of the public sector, believing that it may drive civil servants’ positive work attitudes and behavior by influencing their individual work cognition (Rainey and Steinbauer, 1999). The individual perception of civil servants toward the organizational mission is measured by mission valence, which refers to the degree of perception and recognition of the importance of the organizational mission by employees (Wright, 2007). The higher the mission valence level of civil servants, the more profound their understanding of the value and significance of the organizational mission. They place greater importance on carrying out the mission in their daily work and have a stronger willingness to participate (Wright and Pandey, 2011). At the same time, mission valence can also stimulate individuals’ sense of responsibility, guiding them to complete work tasks with high quality (Wright, 2007). Therefore, as a powerful positive driving force, mission valence not only motivates civil servants to advance the achievement of organizational goals but also guides them to internalize the organizational vision as their own goals. In the context of increasingly diverse public service demands and the need for organizational change, it encourages them to engage in more proactive behaviors that go beyond their job responsibilities (Caillier, 2016). At the same time, according to the Conservation of Resources Theory, innovative work behavior requires individual resource investment (Zhang et al., 2024). Mission valence can be seen as a positive energy resource that provides support for civil servants to engage in innovative work behavior (Guerrero and Chênevert, 2021). In addition, with the nationwide implementation of a series of theme-based education activities centered on “stay true to the Party’s original aspiration and founding mission,” mission-oriented educational activities have led civil servants to develop a deeper understanding and awareness of the organizational mission. As a result, their mission valence is strengthened, which may inspire more innovative work behavior. Based on this, studying the impact of civil servants’ mission valence on their innovative work behavior is key to exploring effective organizational incentive pathways in the public sector, thus addressing governance issues in the public sector.

Innovative work behavior in the public sector context is often accompanied by certain risks and challenges. Therefore, for civil servants to engage in innovation, they not only need individual resources but also require a positive work willingness (Xie et al., 2024). In government departments, harmonious passion refers to an individual civil servant’s strong desire to work (Vallerand et al., 2007), which stems from their intrinsic yearning for their work. It enables civil servants to maintain a highly engaged and energetic work state in order to achieve work goals. The Broaden-and-Build Theory of Positive Emotions suggests that an individual’s positive psychological state can broaden their cognition and, through the building effect, provide them with resources in various aspects, including psychological resources (Fredrickson and Joiner, 2002, 2018). In addition, existing research points out that harmonious passion is an important source of resources for individuals in their work (Houlfort et al., 2013). Therefore, the harmonious passion of civil servants may provide the resource support necessary to stimulate their innovative work behavior, effectively promoting their engagement in innovative activities. At the same time, harmonious passion is also influenced by an individual’s work resources (Bajaba et al., 2025). The perception of the importance of the organizational mission by civil servants can provide them with positive cognitive resources (Bosak et al., 2021), meaning that mission valence and civil servants’ harmonious passion may be positively correlated. Based on this, in the context of the public sector, this study attempts to select harmonious passion as a mediating variable to examine its mediating effect in the relationship between mission valence and innovative work behavior of civil servants.

In addition to intrinsic individual factors, the organizational environment in which civil servants operate also influences their psychological cognition and behavior (Belgasm et al., 2025). Existing research has explored the impact of situational factors such as HR flexibility and benevolent leadership on employees’ harmonious passion (Shen et al., 2023; Luu, 2024). Spiritual leadership is a type of leadership that emphasizes value guidance and belief stimulation (Fry, 2003). According to the Conservation of Resources Theory, spiritual leadership can be viewed as an important organizational resource, and thus may affect the work state of civil servants. The development of harmonious passion, in addition to being promoted by an individual’s own cognition, is also influenced by the leadership closely connected to employees in their work (Elsaied, 2024). If leaders focus on value guidance, providing civil servants with continuous spiritual motivation, along with care for their spiritual well-being, it may continuously enhance their intrinsic work passion (Hamzaa et al., 2025). In other words, the impact of mission valence on civil servants’ harmonious passion may vary depending on the leadership style, meaning that spiritual leadership may either enhance or weaken the effect of mission valence on civil servants’ harmonious passion. In addition to leadership factors, the organizational work climate is also an important factor influencing the behavior of civil servants (Steen and Schott, 2019). An organization’s fault-tolerant climate is an employee’s perception of an organizational environment that allows risk-taking behaviors, accepts mistakes as part of the process, and tolerates errors (Chen et al., 2024). According to the Conservation of Resources Theory, the inclusive and supportive work environment created by the organization’s fault-tolerant climate can provide individuals with various resources, including psychological and emotional support. On this basis, to continuously acquire resources, civil servants are more likely to view innovation as an opportunity to obtain resources. In a state of harmonious passion, they are more proactive and bolder in engaging in innovation. Therefore, the strength of the impact of civil servants’ harmonious passion on innovative work behavior may be influenced by the organization’s fault-tolerant climate. Based on this, this study attempts to explore the boundaries of how mission valence affects civil servants’ innovative work behavior by selecting spiritual leadership and the organization’s fault-tolerant climate as situational variables, focusing on two core organizational elements: leadership and organizational climate.

In summary, based on the Conservation of Resources Theory and the Broaden-and-Build Theory of Positive Emotions, this study explores the intrinsic mechanisms through which mission valence affects civil servants’ innovative work behavior. This study may contribute in the following ways: First, it verifies the positive impact of mission valence on civil servants’ innovative work behavior and analyzes the mediating mechanism through harmonious passion between the two. Second, it examines the moderating effects of spiritual leadership and the organization’s fault-tolerant climate in the path through which mission valence influences civil servants’ innovative work behavior via harmonious passion, explaining the interaction between individual and organizational factors in shaping civil servants’ behavior. Therefore, in order to deeply analyze the process mechanisms and boundary conditions through which mission valence influences civil servants’ innovative work behavior, this study constructs a moderated mediation model. The aim is to enrich the theoretical outcomes of mission valence and provide theoretical and practical insights for enhancing civil servants’ harmonious passion and, in turn, promoting their innovative work behavior.

## Literature review and hypotheses

### Mission valence and innovative work behavior

Innovation is a multi-stage, discontinuous process of behavioral activity (Scott and Bruce, 1994). Innovative work behavior of civil servants refers to the process in which civil servants propose, apply, and promote new ideas or technologies in order to improve government operational efficiency and public service quality, focusing on work processes, methods, and existing issues (Miao et al., 2018). Unlike the fundamental innovation traits in the private sector, innovative work behavior in the public sector, as a role-external behavior, is relatively moderate in terms of innovation. It mainly manifests as innovation in ideas and processes (Kruyen and van Genugten, 2017), which is crucial for enhancing the adaptability to change in the public sector (Van der Voet et al., 2016).

Existing research has explored the antecedents of civil servants’ innovative work behavior, finding that, in addition to institutional and organizational factors, the individual-level perception of civil servants toward the organization and their work is a key factor influencing their innovation. For example, challenging or obstructive performance pressure has a double-edged effect on civil servants’ innovative work behavior (Du et al., 2025); the sense of responsibility for change among civil servants significantly positively influences their innovative work behavior (Campbell, 2018; Tran et al., 2023). Similarly, mission valence, as an important cognitive variable for civil servants, may also be one of the key factors influencing their innovative work behavior. Mission valence reflects the subjective judgment and evaluation of civil servants regarding the importance and inherent value of the organizational mission (Rainey and Steinbauer, 1999) and reflects the degree to which the organizational mission motivates civil servants. A high level of mission valence for an individual means that civil servants value the mission of the department they belong to, believing that their daily work carries important social value. As a result, with the goal of more efficiently addressing problems that arise in their work, they are more likely to proactively think, seek new ideas and methods for problem-solving, and put them into practice. Therefore, the higher the mission valence of civil servants, the more likely they are to exhibit innovative work behavior. A small number of existing studies have confirmed this; for example, scholars have verified that mission valence can significantly positively stimulate proactive behaviors such as suggestion behavior in university faculty and role-external behaviors in civil servants (Caillier, 2016; Liu et al., 2024). Based on this, this study hypothesizes that mission valence will encourage civil servants to engage in more innovative work behavior.

The basic principle of the Conservation of Resources Theory is that individuals have a tendency to acquire, build, and preserve resources they consider valuable to reduce the risk of resource depletion (Hobfoll, 1989). This theory is primarily based on the dynamic changes in an individual’s resource stock to explain the motivations behind individual behavior. It is currently widely used to explain psychological and behavioral phenomena of individual employees in organizational contexts (Hobfoll et al., 2018). Therefore, this theory provides an explanatory framework for this study on the impact of mission valence on civil servants’ innovative work behavior. According to the Conservation of Resources Theory, mission valence can be seen as a rich source of psychological energy (Guerrero and Chênevert, 2021), which motivates employees to actively engage in their work. In order to continuously enhance the value of existing resources, they will take actions to actively expand their resource stock. Employees with high mission valence mean that they strongly identify with and support the organizational mission of achieving public value, and thus possess abundant emotional resources such as trust. Based on this, to increase their resource reserves, they are likely to continuously invest resources. Innovative work behavior is an important pathway through which employees achieve the resource enhancement spiral via resource investment (Choi et al., 2021; Xu et al., 2024). Specifically, given that innovation plays an important role in improving administrative efficiency and optimizing organizational operations in the public sector (Miao et al., 2018), civil servants who exhibit innovative work behavior are often recognized and affirmed, and may consequently gain promotion opportunities or other resources. Therefore, this perception stimulates the utilitarian motivation of civil servants. To preserve and expand resources, they are likely to engage in proactive, innovative work behaviors to acquire these resources, thereby driving organizational change. In addition, the Conservation of Resources Theory also suggests that an individual’s work stress can be alleviated by increasing their resource stock, which in turn stimulates their work motivation. In the context of government reform, facing the complexities of daily tasks, civil servants are experiencing an increasing work burden (Miao et al., 2024), leading them to feel a lack of work meaning and low work accomplishment, which in turn makes them prone to job burnout (Kotzé, 2022). The mission valence of civil servants makes them realize the significance of fulfilling the organizational mission through their daily work, thereby achieving social value. In other words, mission valence provides civil servants with abundant psychological resources, reducing emotional exhaustion and enhancing their work well-being (Bosak et al., 2021), thereby stimulating their work motivation and encouraging them to engage in more innovative work behavior. Based on this, the following research hypotheses are proposed:

*H1*: Mission valence has a significant positive impact on civil servants’ innovative work behavior.

### The mediating role of harmonious passion

In the context of the public sector, harmonious passion is manifested as civil servants’ positive attitude toward and willingness to engage in their daily work (Vallerand et al., 2003). This enables them to maintain high energy and a high level of engagement in their work, which contributes to efficiently achieving work goals. Harmonious passion stems from the intrinsic motivation formed when employees internalize their daily work as an important part of their self-identity. Therefore, individual cognition is an important influencing factor (Bouhalleb and Haddoud, 2024). For example, employees’ perceptions of work environment characteristics (Peyton and Zigarmi, 2024) and their self-assessment of ability levels (Thorgren and Wincent, 2013) have been shown to positively influence the development and generation of their harmonious passion. In addition, according to the Conservation of Resources Theory, an individual’s resource stock influences their subsequent resource investment behavior. Individuals with sufficient resources are more confident and capable of investing resources to achieve continuous growth; in contrast, individuals with resource scarcity tend to focus on protecting existing resources to avoid further resource depletion (Hobfoll et al., 2018). Existing research has found that harmonious passion, by providing employees with emotional resources, makes them more willing to seek positive change and initiate proactive transformation, demonstrating higher levels of creativity (Luu, 2021; Xiao et al., 2022; Zhang et al., 2023). Conversely, if employees’ level of harmonious passion is relatively low, they may choose to preserve existing resources rather than invest in new resources to protect their current psychological resources. In addition, under the relatively rigid management system in the public sector, civil servants tend to adopt a more conservative and cautious attitude toward resource investment behaviors such as innovation (Bysted and Hansen, 2015; Bos-Nehles et al., 2017). Therefore, the absence of positive emotional resources may make them more inclined to follow existing rules and regulations rather than easily break through and innovate. Based on this, according to the Conservation of Resources Theory and existing research, this study hypothesizes that the impact of mission valence on civil servants’ innovative work behavior may be mediated by harmonious passion. The relevant analysis is as follows.

Mission valence emphasizes individuals’ positive cognition of the importance of the organizational mission and its associated public value, which helps to promote civil servants’ harmonious passion. Specifically, civil servants with a high level of mission valence are more likely to be strongly inspired and attracted by the organizational mission. As a result, they develop interest and passion for the daily tasks involved in fulfilling the organizational mission, believing that their work in implementing public policies and providing public services carries significant social meaning and public value. This enhances their internal motivation (Wright, 2007), leading to a higher level of harmonious passion. Therefore, this study proposes the following research hypothesis:

*H2*: Mission valence has a significant positive impact on civil servants’ harmonious passion.

Harmonious passion is not only an important driving force for civil servants to overcome difficulties in organizational change, but also a crucial cognitive and emotional resource that drives individuals to engage in innovative work behavior. First, employees with harmonious passion often have stronger dedication and a sense of responsibility in their work (Rai et al., 2024), displaying higher levels of self-efficacy (Cheng, 2024). As a result, they are more willing to invest their energy and time in challenging yet beneficial change activities that align with organizational goals. Second, the positive emotional attributes associated with harmonious passion can directly affect employees’ work state (Guo et al., 2025), making them more likely to experience excitement and vitality rather than anxiety or helplessness when facing difficulties (Klaukien et al., 2013). This, in turn, makes them more inclined to embrace change and challenges (St-Louis et al., 2018), thereby stimulating more novel ideas and creativity. In addition, according to the Broaden-and-Build Theory of Positive Emotions, the positive emotions associated with harmonious passion not only enhance individual resources through their building effect but also improve cognitive flexibility and creativity (Fredrickson, 2001). This also ensures that civil servants engage in innovative work behavior. Therefore, this study proposes the following hypothesis:

*H3*: Harmonious passion has a significant positive impact on civil servants’ innovative work behavior.

Although existing research has gradually started to focus on the issue of transformative innovation among civil servants in the public sector, aiming to improve civil servants’ work innovation levels, the impact mechanism of mission valence on civil servants’ innovative work behavior in the government context still requires further exploration. Demerouti et al. (2001), in the Job Demands-Resources model, indicated that the work environment can be divided into two categories: job demands and job resources. These two types of factors can influence employees’ psychological state, which in turn affects their behavior and outcomes. Based on existing research, it can be seen that, like other positive work states, harmonious passion is a work state that arises when an individual internalizes their work as part of their self-identity. It is characterized by positive psychological traits and serves as an important link between individual cognition and behavior (Forest et al., 2012).

Based on this, this study posits that in the public sector context, harmonious passion is an individual work state that effectively transmits civil servants’ mission valence, which reflects their organizational mission value recognition, thereby enhancing their innovative work behavior. On one hand, civil servants with a high level of mission valence typically have a deep recognition of the social value of their work. They perceive the meaning and importance of their daily tasks, which generates a continuous source of spiritual motivation, forming harmonious passion. On the other hand, civil servants in a strong, harmonious passion state are usually energetic, focused on their work, and willing to take risks. They not only complete their primary duties but also have a strong desire to perform their work better to fulfill the organizational mission they identify with. Therefore, mission valence helps to strengthen civil servants’ positive cognition of the organizational mission, making them willing to internalize its values, thereby enhancing their harmonious passion. At the same time, harmonious passion improves civil servants’ work state, prompting them to proactively and creatively think about how to optimize processes and improve service quality, thus exhibiting more innovative work behavior. Based on the above analysis, this study proposes the following hypothesis:

*H4*: Harmonious passion mediates the relationship between mission valence and civil servants’ innovative work behavior.

### The moderating role of spiritual leadership

As mentioned earlier, mission valence is the subjective cognition of civil servants based on the importance and value of the organizational mission. In addition to being influenced by the internal cognition of mission valence, harmonious passion, as a work emotion and state, is also affected by the external organizational environment (Ho and Astakhova, 2020). Leaders are often seen as representatives of the organization, and their style and behavior tend to have an impact on employees’ work state and emotions (Kousina and Voudouris, 2023). Spiritual leadership refers to a leadership style that affirms and supports employees’ spiritual existence by conveying the organization’s vision, instilling hope and belief, and expressing altruistic love, thereby fulfilling employees’ spiritual needs (Fry, 2003). Research has shown that employees influenced by spiritual leadership tend to exhibit stronger work enthusiasm and initiative (Zhu et al., 2022). Given the motivational nature of mission valence, the guidance and encouragement provided by spiritual leadership align with the organizational motivational characteristics of mission valence, helping civil servants deepen their understanding and recognition of the organizational mission. Based on this, this study posits that spiritual leadership may moderate the effect of mission valence on civil servants’ harmonious passion.

Specifically, first, spiritual leadership paints the vision of the public sector for employees, thereby enhancing their motivation to fulfill the organizational mission, deepening their sense of work meaning and value, and stimulating their intrinsic drive through goals, which in turn strengthens the motivational effectiveness of mission valence. Second, the spiritual support from leadership can effectively foster positive motivation in employees. Spiritual leadership, by focusing on and actively guiding employees’ belief in achieving goals, helps build their confidence, enhances their sense of self-efficacy, and generates positive beliefs in fulfilling the organizational mission. This, in turn, strengthens their willingness to work diligently. Finally, in the face of complex daily administrative tasks, spiritual leadership actively responds to employees’ spiritual needs by expressing respect, care, and support, thereby fulfilling their sense of belonging and, in turn, enhancing their identification with the organizational mission. Based on the principle of reciprocity, civil servants are more likely to invest more in their work as a way to reciprocate the care and support provided by their leaders. Based on this, this study proposes the following hypothesis:

*H5*: Spiritual leadership positively moderates the relationship between mission valence and civil servants’ harmonious passion.

### The moderating role of the organization’s fault-tolerant climate

Innovative work behavior involves trying and exploring, even breaking existing rules. Due to individuals’ limited rationality, the innovation process is often accompanied by the risk of errors (Torugsa and Arundel, 2017). For civil servants, in the context of increasingly strengthened accountability mechanisms and social supervision, mistakes that may arise during the innovation process not only have the potential to waste work resources but also may bring negative consequences to themselves (Choi, 2025). Existing research has found that the emergence of innovative work behavior is influenced by multiple factors, including organizational environment, job characteristics, and individual perceptions (Wynen et al., 2020). Although an increase in harmonious passion may promote civil servants’ innovative work behavior, the inherent risk attributes of innovation, coupled with civil servants’ concerns about the potential negative consequences of deviating from the norm, may hinder the effective transformation of innovation willingness into behavior. Therefore, innovative work behavior exhibits situational dependency characteristics (Chen et al., 2021). In the context of the public sector, the fault-tolerant environment created by the organization, as a key external variable, may influence the innovation willingness and behavior of organizational members. An organization’s fault-tolerant climate refers to civil servants’ psychological perception of their organization’s tolerance for trial-and-error, risk-taking, and mistakes (Liu and Li, 2021). It can be understood as an atmosphere created by government departments through a series of institutional policies in the context of establishing error-correction mechanisms and encouraging civil servants to take responsibility, aimed at reducing penalties for mistakes or losses caused by attempts to innovate. The goal is to enhance the innovation, enthusiasm, and behavior of civil servants, providing them with the necessary opportunities for innovation, i.e., representing “I have the opportunity to innovate.” Therefore, this study posits that differences in the organization’s fault-tolerant climate may moderate the impact of harmonious passion on civil servants’ innovative work behavior.

Specifically, first, harmonious passion reflects the positive psychological state exhibited by civil servants when facing work tasks, allowing them to have abundant psychological energy to perform their work and be inclined to provide public services more efficiently. At the same time, in a high-level fault-tolerant organizational climate, as the organization demonstrates greater tolerance for mistakes, civil servants are less likely to adopt an avoidance attitude due to the potential risk of errors. They also do not overly worry about the accountability consequences of mistakes that may result from innovative work behavior. This allows them, in a positive work state, to be more willing to proactively identify problems in the work process, engage in innovative thinking, generate creative ideas, and implement creative solutions, thereby facilitating the display of innovative work behavior. Secondly, a positive and inclusive organizational error management attitude and behavioral practices not only provide civil servants with institutional security but also ensure that when organizational members face difficulties in innovative work behavior, they can feel support and assistance from colleagues and leaders. This minimizes the likelihood of them abandoning innovative work behavior due to the inability to withstand organizational accountability and social pressure after failure. Finally, according to the Conservation of Resources Theory, individuals tend to continuously acquire resources. Establishing a high level of fault-tolerant climate within the organization can effectively create an inclusive and supportive work environment. This environmental characteristic not only significantly reduces the negative emotional experiences of grassroots civil servants caused by work mistakes (Wang et al., 2020) but also strengthens their sense of belonging and loyalty to the organization. As a result, it enhances their perception of psychological safety, alleviates career stress caused by resource depletion (Wang et al., 2018), and encourages them to acquire more resources in emotional, psychological, skill, and informational aspects. On this basis, civil servants are more likely to view “innovation through trial and error” as an opportunity to acquire resources, thereby becoming more actively engaged in innovation. Conversely, if individuals have a low perception of the organization’s fault-tolerant climate, under the dual influence of a “blame avoidance” mentality and a “seeking stability” tendency, they are more likely to choose conservatism and resist innovation to avoid potential resource depletion, thereby displaying lower levels of innovation willingness. In summary, this study proposes the following hypothesis:

*H6a*: Organization’s fault-tolerant climate positively moderates the relationship between harmonious passion and civil servants’ innovative work behavior.

Building on the integration of the aforementioned hypotheses, this study further hypothesizes that an organization’s fault-tolerant climate can moderate the mediating role of harmonious passion in the relationship between mission valence and civil servants’ innovative work behavior. In a weaker organization’s fault-tolerant climate, civil servants may worry about the mistakes and losses caused by change and innovation. As a result, they may innovate more cautiously, which weakens the impact of harmonious passion on innovative work behavior. Consequently, the positive effect of mission valence on civil servants’ innovative work behavior through harmonious passion will also be weakened. In contrast, in a stronger organization’s fault-tolerant climate, civil servants feel supported by the organization in terms of change and are more tolerant of mistakes. This reduces their pressure to avoid blame. In a state of harmonious passion, they are more likely to engage in innovative work behavior. As a result, the impact of harmonious passion on innovative work behavior is strengthened, and consequently, the positive effect of mission valence on innovative work behavior through harmonious passion is also enhanced. Based on this, this study proposes the following hypothesis:

*H6b*: Organization’s fault-tolerant climate positively moderates the mediating role of harmonious passion in the relationship between mission valence and civil servants’ innovative work behavior.

**FIGURE 1:**
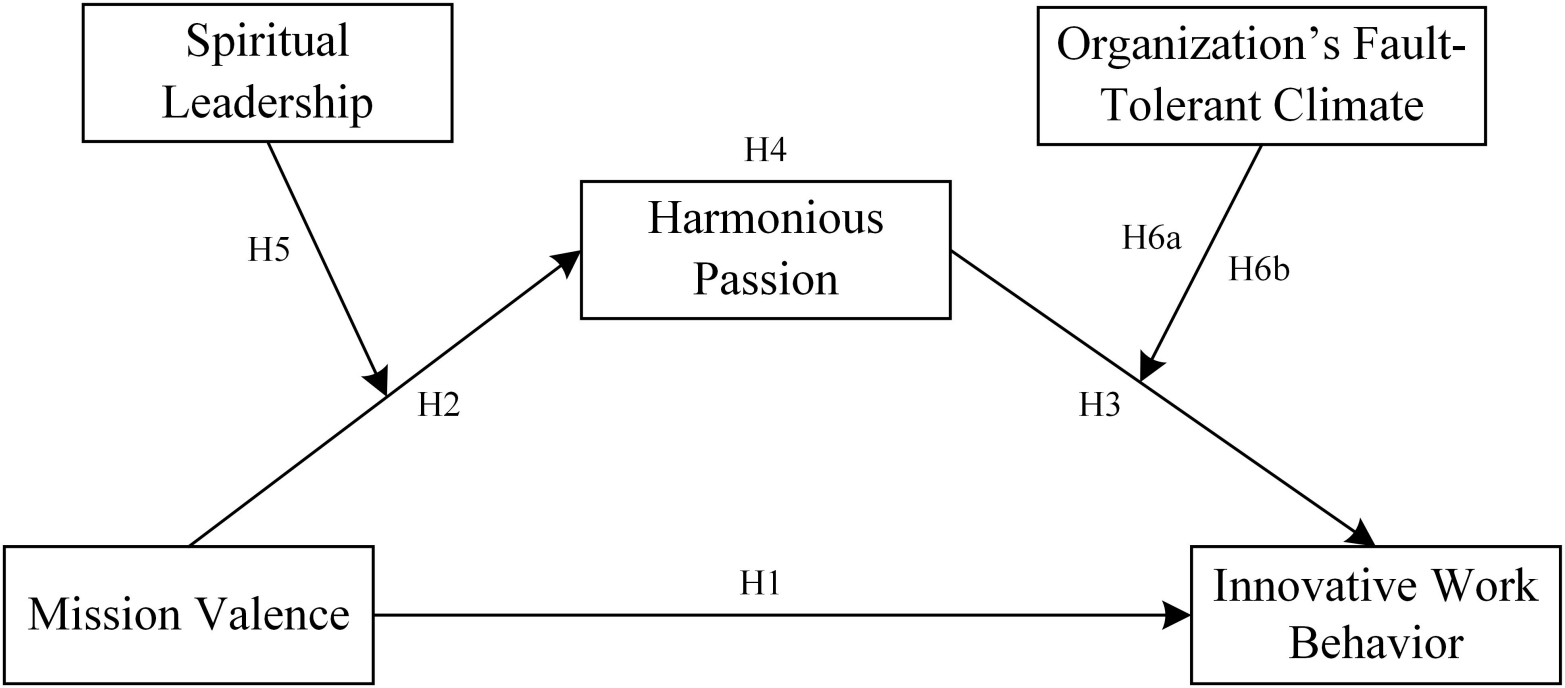
The research model of the study.

In summary, this study proposes the following moderated mediation model, as shown in Figure 1.

## Materials and methods

### Sample and data collection

Before the formal survey, semi-structured interviews were conducted with MPA (Master of Public Administration, MPA) students from our college who were also civil servants. These interviews not only explored their perceptions of their own careers and the organizational mission but also focused on reviewing all the items in the questionnaire to ensure that the wording was consistent with the actual context of Chinese government departments and aligned with civil servants’ understanding. Ambiguous or obscure terms were revised accordingly. The formal survey was conducted using a combination of online and on-site distribution methods. The online distribution relied on alumni resources, with current civil servant alumni and friends invited to distribute electronic questionnaires to their colleagues in their departments through a snowball sampling method. On-site distribution was primarily facilitated by MPA students from the school, who distributed the questionnaires during class sessions. The survey participants were primarily government civil servants, including those from departments such as health, civil affairs, education, and taxation.

To control for common method bias and social desirability effects, this study employed a two-wave survey with a 6-week interval. Such temporal separation is an effective procedural remedy for common method bias, as it helps break transient mood effects, reduce priming influences, and mitigate consistency motives, and has been widely applied in organizational behavior research (Podsakoff et al., 2003). The 6-week interval represents a commonly adopted compromise in organizational behavior studies, effectively separating measurements while minimizing sample attrition due to overly long intervals (Fuller et al., 2016). In addition, it helps break down the measurement tasks, alleviating respondent fatigue that may arise from completing all items in a single session.

The first stage of the survey mainly collected respondents’ basic information, mission valence, and harmonious passion. A total of 530 questionnaires were distributed, with 502 returned. After excluding questionnaires with lengthy response times and many missing answers, 487 valid samples were obtained. The second stage of the survey mainly collected respondents’ innovative work behavior, spiritual leadership, and the organization’s fault-tolerant climate. A total of 520 questionnaires were distributed, with 507 returned. After excluding invalid responses, 493 valid samples were obtained. Finally, a two-stage data matching method was used to filter the survey data, successfully matching 456 samples. The valid sample recovery rate was 86%, meeting the basic validity requirements for empirical research. In terms of data processing, this study used listwise deletion to handle missing values. During the data cleaning process, questionnaires with excessive missing data were discarded, and the final sample of 456 responses used for analysis had no missing values across all research variables.

In the valid sample of this study (see Table 1), by gender, 220 were male, accounting for 48.2%, and 236 were female, accounting for 51.8%. In terms of age distribution, the majority were between 26 and 35 years old, with 298 people, accounting for 65.4%. Regarding education level, 20 had an associate degree or below, accounting for 4.4%, 312 had a bachelor’s degree, accounting for 68.4%, and 124 had a master’s degree or higher, accounting for 27.2%. In terms of work experience, 34 had < 1 year, accounting for 7.5%, 192 had 1–3 years, accounting for 42.1%, 116 had 4–6 years, accounting for 25.4%, 61 had 7–10 years, accounting for 13.4%, and 53 had more than 10 years, accounting for 11.6%.

**TABLE 1:** Basic information of the survey respondents (*N* = 456).

Characteristic	Classification	Frequency	Percentage(%)
Gender	Male	220	48.2
	Female	236	51.8
Age	25 years old or below	88	19.3
	26–35	298	65.4
	36–45	48	10.5
	46–55	19	4.2
	56 years old or above	3	0.7
Education	Associate degree or below	20	4.4
	Bachelor’s degree	312	68.4
	Master’s degree or above	124	27.2
Tenure	1 year or below	34	7.5
	1–3 years	192	42.1
	4–6 years	116	25.4
	7–10 years	61	13.4
	10 years or above	53	11.6

### Variable measurement

The five variables in this study were measured using established scales from authoritative journals, which have good reliability and validity. These scales have also been well tested in Chinese domestic studies, demonstrating their appropriateness in the Chinese context. The main variables were measured using a five-point Likert scale, where “1” indicates strongly disagree or not applicable, and “5” indicates strongly agree or applicable. The scores for each item were selected by the respondents based on their personal judgment.

For the measurement of mission valence, this study used the scale developed by Wright (2007), which includes three items, such as “This division provides valuable public services.” The Cronbach’s α coefficient for this scale in this study is 0.91.

The measurement of harmonious passion was adapted from a part of the scale developed by Vallerand et al. (2003), consisting of 7 items such as “My work is well aligned with my life.” The Cronbach’s α coefficient for this scale in this study is 0.93.

The measurement of innovative work behavior of civil servants was adapted from the scale developed by Bysted and Hansen (2015), consisting of 6 items such as “I am creating new ideas for improvements.” The Cronbach’s α coefficient for this scale in this study is 0.94.

The measurement of spiritual leadership was adapted from the scale developed by Fry et al. (2005), which includes 17 items such as “I understand and am committed to my organization’s vision.” The Cronbach’s α coefficient for this scale in this study is 0.97.

The measurement scale for the organization’s fault-tolerant climate was developed by Liu and Li (2021) specifically for the Chinese government context. It includes 14 items, such as “My supervisor will not blame employees for unavoidable mistakes in their work.” The Cronbach’s α coefficient for this scale in this study was 0.96.

At the same time, based on existing research (Hammond et al., 2011; Uppathampracha and Liu, 2022), this study selects gender, age, education, and tenure as control variables. Gender, a categorical variable, is recoded using dummy variables, with 1 representing male and 2 representing female. Additionally, age, education, and tenure are treated as continuous variables. Additionally, all items in the scales used in this study were positively scored, with no reverse scoring involved.

## Results

### Common method bias

This study first employed the Harman single-factor test to examine the level of common method variance in the sample data. Principal component analysis extracted five factors, with an overall explanatory rate of 71.57%. In the unrotated solution, the first factor explained 47.06% of the variance, which is below the critical threshold of 50% (Podsakoff et al., 2003; Fuller et al., 2016). Next, confirmatory factor analysis (CFA) was conducted on all measurement items using AMOS 24.0. The results showed that the five-factor model provided a better fit than other factor models, with all major fit indices meeting the required thresholds, as shown in Table 2. Specifically, χ^2^/df = 2.00 (<3), NFI (Normed Fit Index), IFI (Incremental Fit Index), TLI (Tucker-Lewis Index), and CFI (Comparative Fit Index) all > 0.9, RMSEA (Root Mean Square Error of Approximation) = 0.047 (< 0.05), and SRMR (Standardized Root Mean Square Residual) = 0.039 (MacCallum et al., 1996; Hu and Bentler, 1999). At the same time, this study employed the control of the unmeasured latent method factor approach to test for common method bias in the data. After adding the common method factor, the model fit was slightly improved, but the changes in various indicators (ΔRMR = 0.009, ΔNFI = 0.006, ΔIFI = 0.004, ΔTLI = 0.002, ΔCFI = 0.004, ΔRMSEA = 0.001) were all < 0.02, indicating that there is no significant common method bias in the data of this study. Additionally, a comparison of the five confirmatory factor models reveals that these five variables are mutually independent and exhibit good discriminant validity.

**TABLE 2:** Model fit results (*N* = 456).

Model	Factor	χ ^2^	*df*	χ ^2^/*df*	RMSEA	RMR	NFI	IFI	TLI	CFI
5-Factor	MV;IB;HP;SL;FC	2047.555	1,024	2.000	0.047	0.050	0.901	0.948	0.945	0.948
4-Factor	MV;IB;HP;SL + FC	4124.895	1,028	4.013	0.081	0.089	0.801	0.843	0.834	0.842
3-Factor	MV;IB;HP + SL + FC	6273.410	1,031	6.085	0.106	0.153	0.697	0.734	0.720	0.733
2-Factor	MV;IB + HP + SL + FC	8045.491	1,033	7.788	0.122	0.169	0.611	0.644	0.626	0.643
1-Factor	MV + IB + HP + SL + FC	8705.461	1,034	8.419	0.128	0.170	0.580	0.610	0.591	0.609

MV, Mission valence; IB, Innovative work behavior; HP, Harmonious passion; SL, Spiritual leadership; FC, Organization’s fault-tolerant climate.

### Descriptive statistics and correlation analysis

This study uses SPSS 27.0 for descriptive statistical analysis, and the results are shown in Table 3. First, we conducted a multicollinearity test. In the first step, we examined the correlation coefficients between the main variables (as shown in Table 3). The results showed that all correlation coefficients were below 0.80 (the highest being *r* = 0.745), which initially suggests that multicollinearity is not a serious issue. In the second step, to further confirm this, we calculated the variance inflation factor (VIF) for the independent variables in all regression models. The results showed that all VIF values were below 3.4 (ranging from 1.430 to 3.385), far below the critical threshold of 10 (and the tolerance values were all above 0.10), indicating that there is no multicollinearity problem among the variables in this study.

**TABLE 3:** Means, standard deviations, and correlations among study variables (*N* = 456).

Variables	Mean	SD	1	2	3	4	5
1. MV	3.777	1.087	1				
2. IB	3.715	0.964	0.651***	1			
3. HP	3.734	0.950	0.744***	0.745***	1		
4. SL	3.771	0.921	0.471***	0.444***	0.495***	1	
5. FC	3.617	0.905	0.402***	0.401***	0.451***	0.693***	1

MV, Mission valence; IB, Innovative work behavior; HP, Harmonious passion; SL, Spiritual leadership; FC, Organization’s fault-tolerant climate;

*** *p* < 0.001.

Second, we conducted a normality test. Following the criteria proposed by Kline (2023), data distribution is considered approximately normal when the absolute value of skewness is < 2 and the absolute value of kurtosis is < 7. In this study, the skewness and kurtosis values of all variables met the standard, indicating that the data are normally distributed.

Finally, we conducted a preliminary analysis of the correlations between the variables. The results indicate that mission valence is significantly positively correlated with innovative work behavior (*r* = 0.651, *p* < 0.001), suggesting a strong correlation between the two. At the same time, mission valence is significantly positively correlated with harmonious passion (*r* = 0.744, *p* < 0.001), and harmonious passion is also significantly positively correlated with innovative work behavior (*r* = 0.745, *p* < 0.001). The correlations among these three variables provide preliminary support for Research Hypotheses 1, 2, and 3. In addition, spiritual leadership is significantly positively correlated with mission valence (*r* = 0.471, *p* < 0.001), harmonious passion (*r* = 0.495, *p* < 0.001), and innovative work behavior (*r* = 0.444, *p* < 0.001). Moreover, organizational fault-tolerant climate is also significantly positively correlated with mission valence (*r* = 0.402, *p* < 0.001), harmonious passion (*r* = 0.451, *p* < 0.001), and innovative work behavior (*r* = 0.401, *p* < 0.001), providing a foundation for further regression model testing.

### Hypothesis testing

#### Mission valence and innovative work behavior: the mediating role of harmonious passion

This study uses a stepwise regression method to analyze the relationship between mission valence and civil servants’ innovative work behavior, and the results are shown in Table 4. Model 4 indicates that mission valence has a significant positive effect on innovative work behavior (β = 0.573, *p* < 0.001). After considering the control variables, mission valence explains 41.3% of the variance in innovative work behavior, suggesting that the higher the level of mission valence, the more pronounced the civil servants’ innovative work behavior. Hypothesis 1 is therefore supported.

**TABLE 4:** Mediation effect analysis of harmonious passion (*N* = 456).

Variables	HP		IB			
	Model 1	Model 2	Model 3	Model 4	Model 5	Model 6
Gender	−0.196*	−0.133*	−0.193*	−0.137*	−0.045	−0.059
Age	−0.016	0.009	−0.065	−0.043	−0.053	−0.048
Education	−0.094	−0.003	−0.112	−0.032	−0.041	−0.030
Tenure	0.050	0.002	0.050	0.008	0.013	0.007
MV		0.647***		0.573***		0.193***
HP					0.752***	0.588***
*R* ^2^	0.018	0.559	0.017	0.430	0.557	0.578
*F*	2.046	114.028***	1.971	67.891***	113.298***	102.607***
Δ*R*^2^	0.018	0.541	0.017	0.413	0.540	0.148

MV, Mission valence; IB, Innovative work behavior; HP, Harmonious passion.

*p < 0.05,

***p < 0.001.

Model 2 indicates that mission valence has a significant positive effect on harmonious passion (β = 0.647, *p* < 0.001). After considering the control variables, mission valence explains 54.1% of the variance in harmonious passion, suggesting that the stronger the civil servants’ mission valence, the higher their level of harmonious passion. Hypothesis 2 is therefore supported. Model 5 shows that harmonious passion has a significant positive effect on civil servants’ innovative work behavior (β = 0.752, *p* < 0.001). After considering the control variables, harmonious passion explains 54% of the variance in innovative work behavior, suggesting that the higher the level of harmonious passion, the more likely civil servants are to exhibit innovative work behavior. Hypothesis 3 is therefore supported.

To examine the mediating effect of harmonious work passion, this study employed the bootstrapping method proposed by Hayes (2013). Using the PROCESS macro for SPSS 27.0, we conducted 5,000 bootstrap resamples to test the significance of the indirect effects. This method assesses mediation by constructing confidence intervals to directly evaluate the statistical significance of the indirect effect. As shown in Table 4, Hypotheses 1, 2, and 3 were supported. The bootstrap test results indicated that the mediating (i.e., indirect) effect of harmonious work passion was 0.381, with a 95% confidence interval of [0.269, 0.503], which does not include zero. This finding demonstrates that harmonious work passion exerts a significant mediating effect between mission valence and civil servants’ innovative work behavior, thus supporting H4. In addition, this study conducted a supplementary test using the mediation analysis procedure proposed by Baron and Kenny (1986). As shown in Model 6, when both mission valence and harmonious work passion were entered into the regression equation simultaneously, the direct effect of mission valence on innovative work behavior remained significant (β = 0.193, *p* < 0.001), but its coefficient decreased from 0.573 in Model 4 to 0.193. This indicates that harmonious work passion plays a partial mediating role in the relationship between mission valence and innovative work behavior, providing further support for Hypothesis 4.

#### Moderating effect of spiritual leadership

**FIGURE 2:**
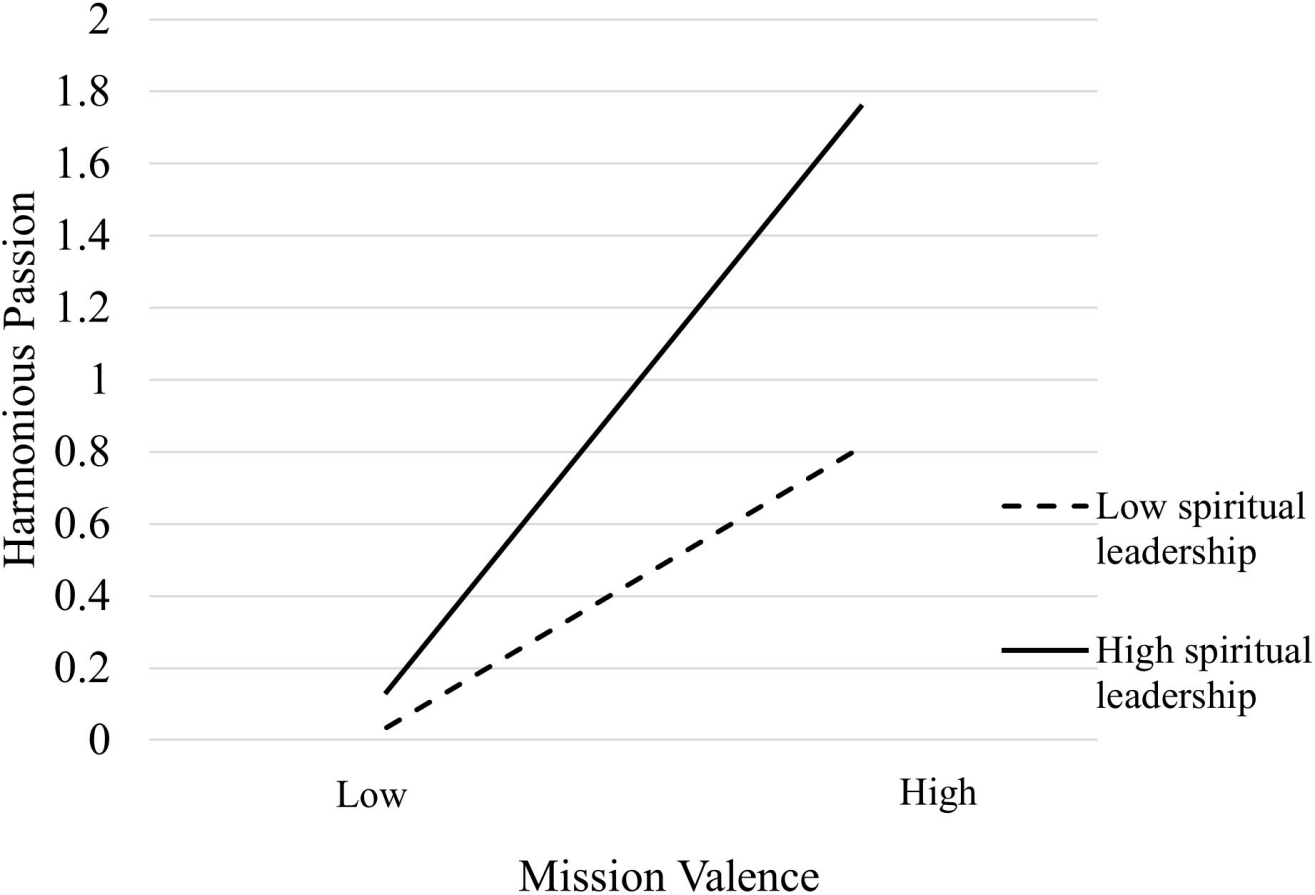
Moderating effect diagram of spiritual leadership.

This study uses SPSS 27.0 hierarchical regression to test the moderating effect of spiritual leadership on the relationship between mission valence and harmonious passion. In the first step, the regression of harmonious passion on control variables is conducted. In the second step, the regression of mission valence on harmonious passion is performed. In the third step, the moderating variable, spiritual leadership, is added. In the fourth step, the interaction term between mission valence and spiritual leadership, after centering, is included. The results of the hierarchical regression are shown in Table 5. The interaction term between mission valence and spiritual leadership has a regression coefficient of 0.212 (*p* < 0.001), and Δ*R*^2^ is 0.071. This indicates that the interaction term accounts for 7.1% of the variance in harmonious passion, suggesting that spiritual leadership positively moderates the impact of mission valence on harmonious passion. Hypothesis 5 is therefore supported. At the same time, following the approach of Aiken et al. (1991), this study plotted the interaction effect using one standard deviation above and below the mean (as shown in Figure 2). The results indicate that the higher the level of spiritual leadership, the stronger the positive impact of mission valence on civil servants’ harmonious passion.

**TABLE 5:** The moderating effect of spiritual leadership (*N* = 456).

Variables	HP			
	Model 1	Model 2	Model 3	Model 4
Gender	−0.196*	−0.133*	−0.142*	−0.123*
Age	−0.016	0.009	−0.002	−0.051
Education	−0.094	−0.003	−0.008	−0.026
Tenure	0.050	0.002	0.008	0.017
MV		0.647***	0.569***	0.600***
SL			0.194***	0.261***
MV × SL				0.212***
*R* ^2^	0.018	0.559	0.586	0.657
*F*	2.046	114.028***	106.097***	122.706***
Δ*R*^2^	0.018	0.541	0.028	0.071

MV, Mission valence; HP, Harmonious passion; SL, Spiritual leadership.

*p < 0.05,

***p < 0.001.

#### Moderating effect of organization’s fault-tolerant climate

**FIGURE 3:**
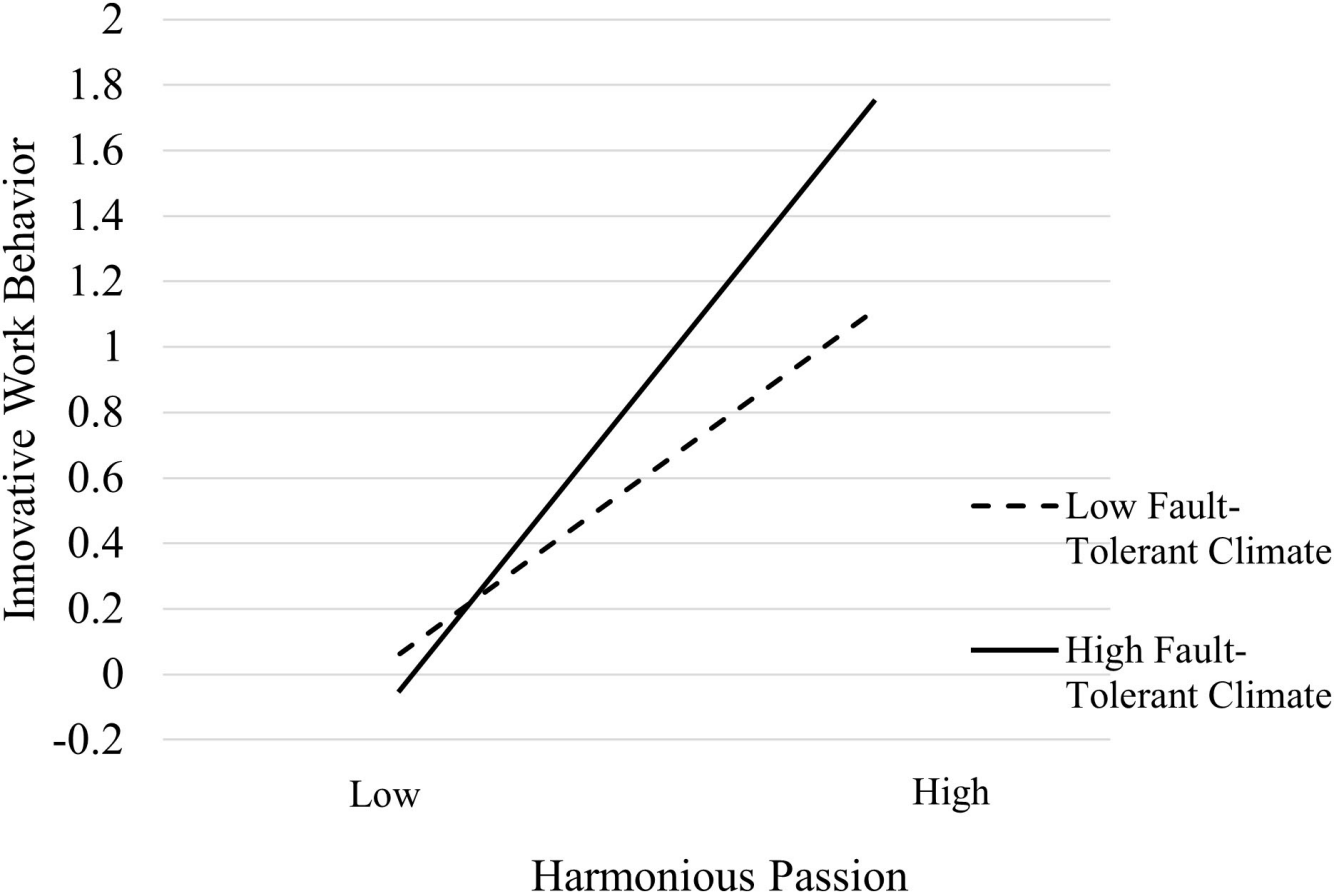
Moderating effect diagram of organization’s fault-tolerant climate.

To test the moderating effect of the organization’s fault-tolerant climate, this study also uses hierarchical regression to examine how the organization’s fault-tolerant climate moderates the effect of harmonious passion on innovative work behavior. The first step is to regress the control variables on innovative work behavior; the second step is to regress harmonious passion on innovative work behavior; the third step adds the moderating variable, the organization’s fault-tolerant climate; and the fourth step introduces the interaction term between harmonious passion and the organization’s fault-tolerant climate after centering. The hierarchical regression results are shown in Table 6. The regression coefficient for the interaction term between harmonious passion and the organization’s fault-tolerant climate on innovative work behavior is 0.188 (*p* < 0.001), and the Δ*R*^2^ is 0.035. This indicates that the interaction term accounts for an additional 3.5% of the variation in innovative work behavior, suggesting that the organization’s fault-tolerant climate positively moderates the effect of harmonious passion on innovative work behavior. Hypothesis 6a is therefore supported. At the same time, this study also followed Aiken et al. (1991) approach by plotting the data with one standard deviation above and below the mean (as shown in Figure 3). The plot demonstrates the moderating effect of the organization’s fault-tolerant climate on the relationship between harmonious passion and innovative work behavior. It shows that as the level of the organization’s fault-tolerant climate increases, the positive impact of harmonious passion on civil servants’ innovative work behavior becomes significantly stronger.

**TABLE 6:** The moderating effect of organization’s fault-tolerant climate (*N* = 456).

Variables	IB			
	Model 1	Model 2	Model 3	Model 4
Gender	−0.193*	−0.045	−0.059	−0.059
Age	−0.065	−0.053	−0.048	−0.070
Education	−0.112	−0.041	−0.046	−0.038
Tenure	0.050	0.013	0.019	0.037
HP		0.752***	0.712***	0.711***
FC			0.091*	0.132***
HP × FC				0.188***
*R* ^2^	0.017	0.557	0.563	0.598
*F*	1.971	113.298***	96.379***	95.248***
Δ*R*^2^	0.017	0.540	0.006	0.035

IB, Innovative work behavior; HP, Harmonious passion; FC, Organization’s fault-tolerant climate.

*p < 0.05,

***p < 0.001.

To further examine the moderated mediation effect, this study employed Hayes’ SPSS PROCESS macro and conducted a bootstrap test with 5,000 resamples (Hayes, 2013). The detailed analysis results are shown in Table 7. When the organization’s fault-tolerant climate is low, the indirect effect of mission valence on innovative work behavior through harmonious passion is 0.300, with a confidence interval of [0.170, 0.446], which does not contain 0. When the organization’s fault-tolerant climate is at a medium level, the indirect effect of mission valence on innovative work behavior through harmonious passion is 0.396, with a confidence interval of [0.285, 0.516], which does not contain 0. When the organization’s fault-tolerant climate is high, the indirect effect of mission valence on innovative work behavior through harmonious passion is 0.491, with a confidence interval of [0.361, 0.627], which does not contain 0. It can be seen that at all three levels of the organization’s fault-tolerant climate, the mediating effect of harmonious passion is significant. Furthermore, as the level of the organization’s fault-tolerant climate increases, the mediating effect of harmonious passion becomes stronger, thus confirming that the mediating effect of mission valence on innovative work behavior through harmonious passion strengthens as the organization’s fault-tolerant climate improves. Furthermore, according to the INDEX and other indicators in Table 7, the organization’s fault-tolerant climate moderates the indirect effect of mission valence on innovative work behavior through harmonious passion, with an INDEX value of 0.105 and a confidence interval [0.025, 0.180] that does not include 0. This indicates that the moderating mediating effect of the organization’s fault-tolerant climate is confirmed, and Hypothesis 6b is supported.

**TABLE 7:** Moderated mediation effect testing results (*N* = 456).

FC	Conditional indirect effects				Moderated mediation effects			
	Effect	SE	LLCI	ULCI	INDEX	SE	LLCI	ULCI
Low	0.300	0.070	0.170	0.446	0.105	0.040	0.025	0.180
Middle	0.396	0.060	0.285	0.516				
High	0.491	0.069	0.361	0.627				

FC, Organization’s Fault-Tolerant Climate.

## Discussion

### Theoretical contributions

The first aspect, based on the context of Chinese government departments, this study enriches the research on the antecedents of civil servants’ innovative work behavior. Specifically, by integrating the Conservation of Resources Theory and the Broaden-and-Build Theory of Positive Emotions, this study constructs and validates the positive impact of mission valence and harmonious passion on civil servants’ innovative work behavior, from the perspectives of organizational incentives in the public sector and individual work states of civil servants.

First, by introducing mission valence, a unique cognitive variable in the public sector, into the study of the antecedents of innovative work behavior, this study verifies the driving mechanism of mission valence on civil servants’ innovative work behavior. This finding aligns with the conclusions of existing related research (Zhang and Wang, 2021) and responds to studies on how mission valence stimulates civil servants’ extra-role behaviors (Caillier, 2016; Liu et al., 2024). At the same time, it also demonstrates that organizational identification can have a positive impact on both individual and organizational development in the public sector context.

Second, the positive impact of harmonious passion on civil servants’ innovative work behavior partially echoes the conclusions of related studies on how work passion stimulates innovative work behavior among civil servants (Zhang et al., 2024; Farooqi et al., 2025).

Finally, this research provides theoretical support for the “stay true to the Party’s original aspiration and founding mission” themed education campaign to enhance civil servants’ spirit of transformation and innovation. It also extends and supplements previous studies on the influencing factors of civil servants’ innovative work behavior, offering valuable reference for future research. In addition, this study responds to the call in existing research to explore the individual cognitive factors influencing civil servants’ innovative work behavior (Eldor, 2017).

It is worth noting that the findings of this study not only show statistical significance but also carry substantial practical significance. From the perspective of effect size, both mission valence’s impact on harmonious passion (β = 0.647, Δ*R*^2^ = 0.541) and harmonious passion’s influence on innovative work behavior (β = 0.752, Δ*R*^2^ = 0.540) exhibit very strong explanatory power. This indicates that mission valence and harmonious passion are not merely statistically correlated variables; rather, they are key factors driving civil servants’ innovative work behavior in practice, with high predictive power for the variation in the outcome variables.

The second aspect, this study verifies the partial mediating role of harmonious passion between mission valence and civil servants’ innovative work behavior. Based on the Broaden-and-Build Theory of Positive Emotions, this research introduces harmonious passion as a variable and confirms its partial mediating role in the relationship between mission valence and innovative work behavior. This reveals a key transmission mechanism, from individual “cognitive resources” to “emotional states” and then to “behavioral outcomes,” providing a new explanation for understanding the psychological mechanism through which mission valence stimulates civil servants’ innovative work behavior.

In addition, this study responds to the call from researchers to analyze the psychological mechanisms behind civil servants’ behavior (Kidron and Vinarski-Peretz, 2024; Yu et al., 2024). More importantly, it partially validates that, in the public sector context, individuals’ emotional mechanisms serve as an effective bridge connecting cognition and behavior, which carries significant transmission implications. This finding resonates with existing research on harmonious passion (Cheng, 2024).

The third aspect, the effect of mission valence on harmonious passion, is influenced by spiritual leadership. The mechanism through which mission valence impacts harmonious passion is shaped by the organizational context, and its strength may vary across different contexts. As a positive leadership style, spiritual leadership plays a crucial role in the impact of mission valence on harmonious passion. Spiritual leadership positively moderates the relationship between mission valence and harmonious passion, such that the higher the level of spiritual leadership, the stronger the positive effect of mission valence on harmonious passion.

This moderating effect aligns with previous studies on the impact of spiritual leadership on employees’ work passion (Ali et al., 2020; Wang et al., 2021; Anser et al., 2021), suggesting that in the public sector, leadership style has a significant influence on civil servants’ behavior (Iqbal et al., 2021).

From a practical significance perspective, the interaction between spiritual leadership and mission valence explained an additional 7.1% of the variance in harmonious passion (Δ*R*^2^ = 0.071). In the context of moderation effect studies in organizational behavior, this constitutes a moderately strong and significant effect. Practically, this suggests that spiritual leadership is not merely a weak auxiliary factor but plays a substantial role in enhancing the conversion process between mission valence and harmonious passion.

The fourth aspect, this study verifies the moderating role of the organization’s fault-tolerant climate in the relationship between harmonious passion and innovative work behavior, clarifying an organizational context condition for this relationship. The findings show that an organization’s fault-tolerant climate strengthens the positive impact of harmonious passion on innovative work behavior. Additionally, this study also finds that an organization’s fault-tolerant climate positively moderates the mediating effect of harmonious passion between mission valence and innovative work behavior.

This conclusion reveals that civil servants’ innovative work behavior is influenced not only by their harmonious passion but also by their perception of the organization’s fault-tolerant climate. Under different organizational fault-tolerant climate conditions, the strength of the impact of harmonious passion on innovative work behavior varies. It is only when civil servants perceive a high fault-tolerant climate that the positive impact of harmonious passion on innovative work behavior is significantly enhanced, and its mediating effect in the relationship between mission valence and innovative work behavior is strengthened. This identifies a contextual condition for the mechanism through which mission valence enhances civil servants’ innovative work behavior, helping to better understand the path mechanism by which mission valence affects innovative work behavior.

This conclusion responds to existing research on the relationship between an organization’s fault-tolerant climate and civil servants’ innovative work behavior (Elsayed et al., 2023), indicating that in bureaucratic systems, the organizational institutional environment plays a crucial role in influencing civil servants’ innovation (Park and Jo, 2017; Tan et al., 2023). It suggests that civil servants’ motivation cannot rely solely on their work states; external organizational environments may have an even more critical impact. Government departments must create a supportive, fault-tolerant climate and provide civil servants with more supportive resources in order to truly stimulate their innovative work behavior, enabling those who want to innovate to have the courage to do so.

At the same time, this study found that the interaction between organizational forgiveness climate and harmonious passion explained an additional 3.5% of the variance in innovative work behavior (Δ*R*^2^ = 0.035). While this effect size is smaller than that of spiritual leadership, it still holds clear practical significance in organizational context research. It confirms that organizational climate plays a critical role in translating harmonious passion into innovative work behavior, highlighting the practical value of providing employees with room for failure to foster innovation.

### Managerial implications

The first aspect, public sector managers should continuously strengthen the mission valence of civil servants as a strategic lever to stimulate organizational innovation. Mission valence is the fundamental driving force in the new era, motivating civil servants to take responsibility and innovate. The core conclusion of this study clearly reveals the internal path of this influence: mission valence can significantly promote innovative work behavior by enhancing individuals’ harmonious passion. Therefore, when implementing the “stay true to the Party’s original aspiration and founding mission” themed education campaign, government departments at all levels should focus on closely aligning macro national strategies and reform goals with the daily duties of civil servants.

Through activities such as organizing lectures by outstanding representatives and promoting advanced role models rooted in the grassroots, the ideological and political construction should be continuously deepened (Zhao and Wu, 2020). The fundamental goal is to foster a deep value recognition of the core mission of “serving the people” among civil servants (Wright, 2007; Sheng and Fan, 2022), enhancing their sense of professional honor and historical responsibility (Willems et al., 2021; Pedersen et al., 2024). According to the Conservation of Resources Theory, this serves to inject civil servants with fundamental and lasting psychological resources (Jia et al., 2024). This, in turn, can be effectively transformed into intrinsic, energizing, harmonious passion and ultimately manifested in outward behavior as innovative work behavior characterized by a willingness to break with conventions and explore practical solutions.

The second aspect is important to focus on, stimulating and cultivating civil servants’ harmonious passion for work. Harmonious passion is the key internal force that drives civil servants to transform their inner identification into innovative practices. The findings of this study indicate that harmonious passion is not only the core mediating mechanism through which mission valence influences civil servants’ innovative work behavior, but its release is also influenced by the organizational context. Therefore, public sector managers should strive to create an organizational environment that fosters and sustains work passion. To achieve this, leaders’ organizational management practices should be intentionally focused on nurturing such an environment.

Specifically, first, at the level of job design, efforts can be made to grant civil servants greater autonomy in their work, enhancing task completeness and feedback on the social value of their work. Existing research has confirmed that such autonomy and flexibility are key job resources for stimulating employees’ intrinsic motivation and work passion (Slemp et al., 2021). This would allow them to more genuinely experience the meaning and sense of accomplishment in their work (Park and Jang, 2017; Autin et al., 2022), thereby nurturing their harmonious passion for work.

Second, in terms of resource support, it is essential to ensure the provision of ample training opportunities, necessary information channels, and technical tools. According to the Conservation of Resources Theory, these key job resources can effectively prevent energy depletion caused by obstacles and resistance (Hobfoll et al., 2018), allowing civil servants to invest more of their psychological energy into the work they are passionate about.

Finally, in terms of human resource management, the organization’s recruitment, assessment, and promotion mechanisms should be appropriately tilted toward individuals who demonstrate intrinsic motivation and work passion (van der Kolk et al., 2019), identifying and motivating those civil servants who genuinely view public service as their professional pursuit. Through these comprehensive measures, the critical bridging role of harmonious passion in the relationship between mission valence and innovative work behavior can be effectively reinforced, ensuring that a sense of mission is truly transformed into an innovative force that drives the organization forward.

The third aspect is crucial to strongly advocate for and strengthen the development of spiritual leadership in the public sector. High levels of spiritual leadership serve as an important organizational guarantee for effectively stimulating civil servants’ intrinsic work passion through mission identification. This study found that when the level of spiritual leadership within a department is high, the positive impact of mission valence on harmonious passion is significantly enhanced. Conversely, a lack of spiritually inspiring leadership can hinder or even deplete civil servants’ sense of mission, making it difficult to transform into intrinsic motivation and thereby weakening the motivating effect of mission valence on harmonious passion.

Specifically, first, organizations should provide systematic training for leadership personnel to enhance their ability to construct and communicate the organizational vision. This will help them effectively connect macro policies with the daily work of their subordinates, clearly articulating the social value that daily tasks can generate (Fry, 2003).

Second, leaders must lead by example, demonstrating a firm belief in public service and a passion for serving others, creating a work atmosphere within the team that is characterized by shared aspirations and emotional warmth. As related research has confirmed, leaders’ care and support can effectively stimulate employees’ enthusiasm (Bronkhorst et al., 2015; Zhu et al., 2022). The ultimate goal is to ensure that civil servants’ sense of mission does not remain at an abstract level, but, under the guidance and encouragement of leaders, is translated into intrinsic and vibrant work passion, thus providing a continuous source of psychological energy for subsequent innovative work behavior.

The fourth aspect is essential to cultivate and maintain an organizational fault-tolerant climate that encourages exploration and tolerates failure. This study found that the organization’s fault-tolerant climate significantly moderates the positive relationship between harmonious passion and civil servants’ innovative work behavior. Specifically, in a high fault-tolerant climate, civil servants’ passion for their work enables them to more boldly engage in the exploration and practice of new methods and models. Moreover, the overall indirect effect of mission valence on innovative work behavior through harmonious passion is also strengthened in such an environment.

To achieve this, first, public sector managers need to shift their mindset and take the lead in establishing a value system that embraces “allowing trial and error, encouraging innovation.” They should view mistakes and even failures in the workplace as valuable opportunities for organizational learning and improvement (Wang et al., 2018), rather than the sole reason for accountability.

Second, institutional safeguards should be put in place. For example, in the performance evaluation system, consideration should be given to innovative attempts, risk-taking, and other forward-looking behaviors to avoid a “results-only” mentality (Lin et al., 2023). Previous studies on the Chinese context have already pointed out that such a fault-tolerant climate is a key factor in enhancing civil servants’ innovative behavior (Liu and Li, 2021). Additionally, establishing a mechanism for reviewing failure cases and sharing experiences can create an open, trusting, and psychologically safe organizational environment, enabling civil servants to innovate and take bold actions.

### Limitations and future research

This study has some limitations and shortcomings, which are as follows:

First, while this study confirms the positive impact of mission valence on civil servants’ innovative work behavior, mission valence may have a “double-edged sword” effect. The study does not clarify the potential negative impacts of mission valence on innovative work behavior. Future research could explore, based on relevant theories, scenarios where mission valence may fail to stimulate innovative work behavior in civil servants.

Second, although we conducted pre-survey interviews to culturally adapt the scales to some extent, most of the measurement instruments used in this study were still originally developed in Western contexts. Although these scales have demonstrated good reliability and validity in previous domestic studies, we did not employ a more rigorous translation–back-translation procedure or conduct an independent pilot test to systematically verify their structural validity among Chinese civil servants. Therefore, these scales may still not fully capture the true attitudes and behaviors of civil servants within China’s specific governance context. Future research should aim to develop localized instruments that are better aligned with Chinese administrative practices, in order to deepen the study of civil servants’ organizational behavior.

Third, this study has certain limitations in data collection methods. Although we used a two-stage data collection (with a 6-week interval) and statistically tested for common method bias, all variables in this study were self-reported by employees. This single-source data approach may introduce social desirability bias, meaning that civil servants may tend to overestimate their actual performance when filling out positive-valence scales such as innovative work behavior. Future research should aim to use multi-source data, such as having direct supervisors evaluate innovative work behavior and using department-level aggregate data for organizational forgiveness climate, to further enhance the reliability of research conclusions.

Fourth, the sample for this study was primarily collected using alumni networks, snowball sampling, and convenience sampling, which constitute non-probability sampling methods. While these approaches enhanced the feasibility of data collection, they may have introduced selection bias—for instance, the sample might not be evenly distributed across regions, hierarchical levels, or departments. This, in turn, somewhat limits the generalizability of the study’s findings to the entire population of Chinese civil servants. Future research should consider employing more rigorous random sampling methods to improve sample representativeness.

Fifth, the design of this study is essentially a time-lagged correlation study, which means we must be cautious when inferring causal relationships. Although the two-stage design is methodologically superior to a purely cross-sectional study, it cannot truly establish causality between variables. For example, this study assumes that mission valence affects harmonious work passion, but reverse causality might also be at play: it could be that civil servants who are already passionate about their work are more likely to positively evaluate and identify with the organization’s mission. Additionally, there may be a reciprocal spiraling relationship between the two variables. Therefore, the conclusions of this study should be more rigorously interpreted as “correlation” rather than “causality.” Future research should adopt more stringent longitudinal designs or field experiments to more robustly test the causal links in the model.

Sixth, the theoretical model of this study also allows for alternative explanations. This study primarily constructs the model based on the Conservation of Resources theory and the Broaden-and-Build theory of positive emotions, and validates the mediating role of harmonious work passion. However, this is only one of many potential mechanisms. The relationship between mission valence and innovative work behavior could also involve other cognitive pathways (such as psychological empowerment) or social pathways (such as organizational identification). Additionally, there may be unmeasured confounding variables that influence both the perception of mission and the willingness to innovate. For example, an individual’s Public Service Motivation or Change-Orientation may simultaneously affect their perception of the mission and their innovation propensity. Future research should explore other potential mediating variables or theoretical perspectives (such as Social Identity Theory) to gain a more comprehensive understanding.

## Conclusion

This study, based on the Conservation of Resources Theory and the Broaden-and-Build Theory of Positive Emotions, explores the mechanism through which mission valence influences civil servants’ innovative work behavior. The study finds that: mission valence has a significant positive impact on civil servants’ innovative work behavior; harmonious passion partially mediates the relationship between mission valence and innovative work behavior, effectively transmitting the influence mechanism of mission valence on civil servants’ innovative work behavior; spiritual leadership positively moderates the effect of mission valence on harmonious passion; and organization’s fault-tolerant climate positively moderates the relationship between harmonious passion and civil servants’ innovative work behavior, as well as the indirect effect of mission valence on innovative work behavior through harmonious passion. Specifically, when the organization’s fault-tolerant climate is high, the impact of harmonious passion on innovative work behavior is stronger, and the mediating effect of harmonious passion is also stronger. The research results provide insights into stimulating civil servants’ innovative work behavior. Public sector managers can adopt corresponding measures to enhance civil servants’ mission valence, strengthen their harmonious passion, while also reinforcing spiritual leadership within the organization and fostering an organization’s fault-tolerant climate. These efforts can help drive civil servants’ transformative innovation, contributing to the long-term development of the public sector and the realization of societal benefits.

## Data Availability

The original contributions presented in the study are included in the article/supplementary material, further inquiries can be directed to the corresponding author.
